# Medical outcomes study social support survey (MOS-SSS) in patients with chronic disease: A psychometric assessment

**DOI:** 10.3389/fpsyt.2022.1028342

**Published:** 2023-01-11

**Authors:** Cesar Merino-Soto, Miguel Ángel Núñez Benítez, Miriam Teresa Domínguez-Guedea, Filiberto Toledano-Toledano, José Moral de la Rubia, Claudia I. Astudillo-García, Leonor Rivera-Rivera, Ahidée Leyva-López, Marisol Angulo-Ramos, Omar Arodi Flores Laguna, Gregorio Hernández-Salinas, Jorge Homero Rodríguez Castro, Omar Israel González Peña, Juan Garduño Espinosa

**Affiliations:** ^1^Instituto de Investigación de Psicología, Universidad de San Martín de Porres, Surquillo, Peru; ^2^Instituto Mexicano del Seguro Social, IMSS, Unidad de Medicina Familiar 31, Mexico City, Mexico; ^3^Departamento de Psicología y Ciencias de la Comunicación, Universidad de Sonora, Hermosillo, Sonora, Mexico; ^4^Hospital Infantil de México Federico Gómez, Unidad de Investigación en Medicina Basada en Evidencias, Mexico City, Mexico; ^5^Instituto Nacional de Rehabilitación Luis Guillermo Ibarra Ibarra, Unidad de Investigación Sociomédica, Mexico City, Mexico; ^6^Instituto Nacional de Ciencias e Innovación para la Formación de Comunidad Científica, INDEHUS, Dirección de Investigación y Diseminación del Conocimiento, Mexico City, Mexico; ^7^Facultad de Psicología, Universidad Autónoma de Nuevo León, Monterrey, Mexico; ^8^Servicios de Atención Psiquiátrica (SAP). Secretaría de Salud, Mexico City, Mexico; ^9^Centro de Investigación en Salud Poblacional, Instituto Nacional de Salud Pública (INSP), Cuernavaca, Morelos, Mexico; ^10^Facultad de Ciencias Empresariales y Jurídicas, Universidad de Montemorelos, Montemorelos, Mexico; ^11^Tecnológico Nacional de México/Instituto Tecnológico Superior de Zongolica-Extensión Tezonapa, Heroica Veracruz, Mexico; ^12^División de Estudios de Posgrado e Investigación, Tecnológico Nacional de Mexico/Instituto Tecnologico de Ciudad Victoria, Ciudad Victoria, Tamaulipas, Mexico; ^13^Dirección de Investigación, Hospital Infantil de México Federico Gómez, Instituto Nacional de Salud, Mexico City, Mexico

**Keywords:** chronic disease, MOS-SSS, primary health care, psychometrics, Mexico, psychometric assessment

## Abstract

**Purpose:**

Currently, information on the psychometric properties of the Medical outcomes study-social support survey (MOS-SSS) for patients with chronic disease in primary health care, suggests problems in the dimensionality, specifically predominant unidimensionality in a multidimensional measure. The aim of this study was to determine the internal structure (dimensionality, measurement invariance and reliability) and association with other variables.

**Methods:**

A total of 470 patients with chronic disease from a Family Medicine Unit at the Instituto Mexicano del Seguro Social, IMSS, with a mean age of 51.51 years were included. Participants responded to the Questionnaire of Sociodemographic Variables (Q-SV), SF-36 Health-Related Quality of Life Scale–version 1.1, and MOS-SSS.

**Results:**

Non-parametric (Mokken scaling analysis) and parametric (confirmatory factor analysis) analyses indicated unidimensionality, and three-factor model was not representative. A new 8-item version (MOS-S) was developed, where measurement invariance, equivalence with the long version, reliability, and relationship with the SF-36 were satisfactory.

**Conclusion:**

The MOS-SSS scale is unidimensional, and the shortened version yields valid and reliable scores for measuring social support in patients with chronic disease at the primary health care.

## 1. Introduction

Globally, an increase in the prevalence of non-communicable diseases (NCDs) (1, 2) has been reported, with the most common being diabetes mellitus (DM), systemic arterial hypertension (HAS), osteoarticular diseases and heart disease. In Mexico, the National Health and Nutrition Survey 2018–2019 (3) reported a prevalence of DM of 10.3% and HAS of 18.4%. NCDs are among the leading causes of death worldwide (1, 2) and in Mexico (4, 5); in addition, they are among the main reasons for consultation at the primary health care (6).

At the international level, the Global Health Metrics (7) reported an increase in the burden of disease associated with NCDs due to the years lost due to premature death (YLLD). Such an increase implies a process of gradual and continuous loss of health, affecting performance, independence, functionality and quality of life (8). It has been shown that the number of comorbid medical conditions is closely related to health-related quality of life (HRQoL) (9, 10) and to limitations in people’s ability to perform activities of daily living (ADLs) (11, 12).

All these health-disease processes involve processes of adversity, risk and vulnerability for patients, their families and the health care system (13, 14). However, despite the warning of national and international agencies about the impact of chronic diseases (CDs) in young adults and older adults, no research processes have been developed to characterize the impact of chronic disease on individual, family and sociocultural indicators associated with social support processes (8).

The first studies on social support focused on psychosocial processes and stress (15) and social support as a buffer for stressful life processes (16). It is a construct that encompasses three components: support schema, support relationships, and support transactions (17). It has been defined as the social resources that people perceive as available or actually provided to them by non-professionals in the context of formal support groups and informal helping relationships that serve as an aid in coping with adverse life events and conditions (18–21). Social support is a determinant of health, and it fulfills different emotional, instrumental, informational, and companionship functions (22).

Thus, individuals’ connections with their social environment occur at the community, social network, and intimate relationship levels (23). The social support has been classified into the following categories (24): (1) material help; (2) behavioral assistance; (3) intimate interaction; (4) guidance; (5) feedback; and (6) positive social interaction (25).

Empirical evidence indicates that social relationships can moderate the effects of stress on people’s health and well-being, which impacts their family, social and work environments (16, 26–30), and in fact, associations between social support and mortality risk have been demonstrated (31–34). Moreover, the sources of social support differ cross-culturally (35, 36). Thus, different mechanisms have been identified through which social networks can influence chronic disease management: sharing knowledge, facilitating access to resources, engaging and maintaining productive relationships with network members (37). For example, a follow-up study conducted in women with school-aged children in the context of the COVID-19 pandemic showed how stress was associated with a higher probability of depression, while social support acted as a buffer against the effects of psychosocial stress and protected physical and mental health (38). Another study conducted in older adults suggested that having few social support networks could be a risk factor for reduced physical functioning, which was linked to dependence in at least one of the ADLs and instrumental ADLs (39). However, other studies have reported that supportive behaviors do not have a positive effect on well-being (40) or may even be detrimental to the recipient (41) or the provider (42).

Given the considerable health implications of social support in patients with chronic disease, the need for a psychometrically sound instrument to measure social support in this population at the primary health care level is indicated. Such findings would provide validation of the Medical outcomes study (MOS) scale of social support in patients with chronic diseases at the first level of health care, obtain useful information to generate empirically based interventions aimed at developing and promoting social support resources, and may provide a novel and complementary approach to improve social support outcomes in this population (8). Therefore, the aim of this study was to evaluate the psychometric properties of the MOS in patients with chronic illness, determine the factor structure of the MOS, estimate its internal consistency reliability, and describe the distribution of MOS scores and the level of social support in the sample. It was hypothesized that the MOS social support survey (MOS-SSS) would show adequate psychometric properties.

The main theoretical formulations and recent empirical research results have focused on social integration, perceived social support, received social support and enacted social support (43–45). Regarding the assessment of social support, various generic and specialized measurement instruments have been developed in the international literature for adults and children and have been classified into measures of social integration, perceived social support, received social support and enacted social support (46, 47). These measures include the Family Relationship Index (FRI) (48), Inventory of Social Support Behaviors (ISSB) (49), Social Provisions Scale (SPS) (50), Social Support Network Inventory (SSNI) (51), among others (52–63).

In Mexico, the evaluation and measurement of social support has been carried out through the following measurement instruments: the Adult Social Support Scale (EAS) (64), the Social Support Scale in Family Caregivers of Older Adults (65), the Social Support Scale in Mexican Adults (66), the Social Support Network Scale (SSNS) (67) that has been validated in family caregivers of children with cancer (8), and the Perceived Social Support Scale (MSPSS) that has been validated in informal primary caregivers of cancer patients and presented satisfactory psychometric properties (68).

Although there are several available measurement instruments, Sherbourne and Stewart (69) developed and evaluated a multidimensional, self-administered 19-item Likert scale of social support (Medical outcomes study-social support survey; MOS-SSS) for patients with chronic illness that assesses five dimensions: emotional support (the expression of positive affect, empathetic understanding and the encouragement of expressions of feelings), informational support (the provision of advice, information, guidance or feedback), tangible support (the provision of material or behavioral assistance), positive social interaction (the availability of other people to do fun things with you), and affective support (including expressions of love and affection). The content of the MOS-SSS was constructed to focus on the sources of social support involved in patient well-being (69), and therefore its content validity is supported by the selection process of the literature and conceptually relevant items. The internal consistency for the 5 dimensions was >0.91, and the overall internal consistency was 0.97. This scale has been validated and adapted to multiple countries and languages, specifically for Chinese (70, 71), Taiwanese (72), Australian (73), Canadian and French (74), and Portuguese (75) populations. The Spanish version (76), used in the present study, was validated in primary health care for patients and consists of 20 items. The first question collects information on the size of the social network. The subsequent 19 items collect values referring to four dimensions of functional social support: emotional/informational support (items 3, 4, 8, 9, 13, 16, 17 and 19), tangible support (items 2, 5, 12 and 15), positive social interaction (items 7, 11, 14 and 18) and affective support (items 6, 10 and 20). In the study by Ahumada et al. (76), the factor analysis revealed the existence of 3 factors, which explained 68.72% of the overall variance. Cronbach’s alpha coefficients for the three factors were >0.85. Factor 1 (items 3, 4, 8, 9, 11, 13, 14, 16, 17 and 19) corresponds to emotional/informational support; factor 2 (items 6, 7, 10, 18 and 20) corresponds to affective support; and factor 3 (items 2, 5, 12 and 15) measures instrumental support.

The Spanish version of the MOS-SSS has been adapted and validated in Mexico in two studies. First, in HIV + patients (77), exploratory factor analysis revealed two factors, namely, emotional/informative support and tangible support; these two factors explained 72.22% of the variance, with Cronbach’s alpha values of 0.97 and 0.89, respectively. Three changes were made to the scale: (1) item 2, “someone to help you when you have to be in bed,” was changed to “someone to help you when you have to be sick in bed”; (2) in item 9, “someone to confide in or talk to about yourself and your concerns,” “yourself” was replaced; and (3) item 1, “approximately how many close friends or close family members do you have?” was changed to item 20 (77). Second, in Mexican patients with cardiovascular disease (78), the results showed four factors: emotional/informational support, positive social interaction, instrumental support and affective support. The internal consistency with Cronbach’s alpha was 0.97, and Cronbach’s alpha for the four factors were >0.95; the four factors explained 87.48% of the variance (78).

To explore the influential methodological points in the previous MOS-SSS validation studies, a systematic scoping review was conducted that focused on the properties of the internal structure. The search was made in a generic engine (Google) and specialized engines (PubMed, Google Scholar) with the keywords in Spanish and English: “validity,” “medical outcomes study (MOS),” and “social support.” The inclusion criteria were articles with validity results in any language and year; studies whose complete content could not be retrieved and manuscripts that were not peer reviewed were excluded. Eligible manuscripts were reviewed by one of the coauthors (MAR), with 100% agreement reached. Some of these articles served as sources to search for additional validation articles [i.e., (79–81)]. The results are presented in Table 1, which shows the essential characteristics of the methodology applied and its influence on internal structure decision making.

**TABLE 1:** Review of MOS-SSS validation studies for evidence of internal structure.

References	Country	Items	Factors	Dimensionality	Fact R	*P* total	Equiv/inva	Equiv long/short
Yu et al. (71)	China 110	19	EMI TAN AFF PSI	CFA: ML	Min = 0.88 Max = 0.99	Yes	N.R.	N.Rel.
Westaway et al. (82)	South Afrika 263	19	EMI TAN	PCA: varimax	N.R.	Yes	N.R.	N.Rel.
Shyu et al. (72)	Taiwan 265	19	EMI TAN	EFA: varimax	R = 0.71	No	N.R.	N.Rel.
Alonso et al. (83)	Portugal 101	19	EMI TAN AFF PSI	PCA: varimax CFA: ML	Min = 0.97 Max = 0.99	Yes	N.R.	N.Rel.
Requena et al. (84)	Spain 400	19	EMI TAN AFF	PCA: varimax	N.R.	No	N.R.	N.Rel.
Gjesfjeld et al. (85)	USA 330	12 4	EMI TAN AFF PSI	CFA: ML	N.R.	Yes	N.R.	Yes *R* > 0.90
Espínola and Enrique (86)	Argentina 375	19	EMI TAN AFF	PCA: varimax	N.R.	Yes	N.R.	N.Rel.
Pais-Ribeiro and Ponte (87)	Portugal 225	19	EMI TAN AFF PSI	PCA: varimax	Min = 0.15 Max = 0.60	Yes	N.R.	N.Rel.
Zanini et al. (81)	Brazil 129	19	EMI TAN AFF PSI	EFA: varimax	N.R.	No	N.R.	N.Rel.
Robitaille et al. (88)	Canada 3,131	19	EMI TAN AFF PSI	CFA: N.R.	N.R.	No	Métrica	N.Rel.
Ashing-Giwa and Rosales (89)	320 Multinational	19	EMI TAN AFF PSI	N.R.	N.R.	No	N.R.	N.Rel.
Londoño et al. (90)	Colombia 179	19	EMI TAN AFF PSI	EFA: varimax/oblicua CFA: N.R.	N.R.	No	N.R.	N.Rel.
Moser et al. (91)	USA 3,241	8	EMI TAN	PCA: varimax CFA N.R.	N.R.	No	N.R.	N.R.
Soares et al. (92)	Brazil	6	One dimension	PCA: varimax	N.Rel.	Yes	N.R.	N.Rel.
Wang et al. (70)	China 200	19	EMI TAN AFF PSI		Min = 0.68 Max = 0.89	Yes	N.R.	N.Rel.
Gomez-Campelo et al. (93)	Spain 1,594	8	One dimension	CFA: ULS	N.Rel.	Yes	N.R.	N.R.
Holden et al. (73)	Australia 20,493	6	One dimension	CFA: ADF	N.Rel.	Yes	N.R.	Yes *R* > 0.90
Basurto et al. (77)	Mexico	19	Emoc (14) Tang (5)	PCA: varimax CFA: MLR	N.R.	No	N.R.	N.Rel.
Giangrasso and Casale (94)	Italia 485	19	EMI TAN AFF PSI	CFA: N.R.	Min = 0.46 Max = 0.75	Yes	N.R.	N.Rel.
Conte et al. (95)	USA 505	19	EMI TAN AFF PSI	PCA: N.R. CFA: N.R.	N.R.	No	N.R.	N.Rel.
Higgins et al. (96)	USA 406	8 4	One dimension	CFA: WLSMV	N.Rel.	No	N.R.	N.R.
Norhayati et al. (97)	Malasya 144	16	EMI Tan Pos	CFA: N.R.	Min = 0.39 Max = 0.86	No	N.R.	N.Rel.
Yu et al. (98)	China 200	19	Emoc (14) Tang (5)	EFA: oblicua		Yes	N.R.	N.R.
Togari and Yokoyama (99)	Japan 2,052	8	Instrum (4) Emoc (4)	PCA: promax	N.R.	Yes	N.R.	N.Rel.
Zanini and Peixoto (80)	Brazil 998	19	EMI TAN AFF PSI	CFA: ML	Min = 0.41 Max = 0.73	No	N.R.	N.Rel.
Priede et al. (100)	Spain 128	19	E-I-SI Instru Afec	PCA: varimax	N.R.	No	N.R.	N.Rel.
Margolis et al. (79)	USA 199	19	One dimension	CFA: WLSMV	Min = 0.88 Max = 0.96	No	N.R.	N.Rel.
Yilmaz and Bozo (101)	Turkey	19	EMI TAN AFF PSI	EFA: varimax	N.R.	No	N.R.	N.Rel.
Martin-Carbonell et al. (102)	Colombia 463	19	EMI TAN AFF PSI	CFA: ULS	Min = 0.77 Max = 0.95	Yes	No	N.Rel.
Navarrete et al. (78)	Mexico 229	19	EMI TAN AFF PSI	PCA: N.R. CFA: ML	Min = 0.59 Max = 0.75	No	N.R.	N.Rel.
Bavarsad et al. (103)	Iran 420	5	Inst (2) Emoc (3)	PCA: varimax CFA: ML	0.55	Yes	No	No

EFA, exploratory factor analysis; PCA, principal components analysis; CFA, confirmatory factor analysis; varimax and oblique, types of rotations; ML WLSMV, ULS, ADF, MLR, estimators; Fact R, interfactor correlations; P total, total score computed; Equiv/inva, measurement equivalence/invariance; Equiv long/short, equivalence between long and short forms; EMI, emotional/informational support; TAN, tangible support; AFF, affective support; PSI, positive social interaction; N.R., not reported; N.Rel., not relevant.

The results of the scoping review indicated that the most tested model was the correlated factors model, and although this model accommodates the generalized tendency of the most used model in psychometric research (104), there are other reasonable models that can be solved in the assessment of the dimensionality of the MOS-SSS, given the evidence of factors with high or very high correlations with each other (Table 1, under the Fact R heading). Along similar lines, model comparisons were almost absent with the exception of a few studies [e.g., (79, 85, 94)], given that they directly tested the correlated factors model, and the confirmatory methodology was not exploited to verify other reasonably competitive models with support in antecedent research.

On the other hand, in this review, it was also found that inter-factor or inter-observed score correlations were rarely reported, even though these psychometric parameters are important for assessing the discriminative validity of the dimensions, and it is usual for confirmatory factor analysis (CFA) to report it (unless explicitly not estimated). In the studies where interfactor correlations were reported [including the study by Sherbourne and Stewart (69)], these tended to show high values, to a degree that raises suspicions about the conceptual discrimination of the dimensions; furthermore, with the exception of a few studies [e.g., (87)], the discriminative validity of the dimensions in the remaining studies is a matter of reasonable doubt. There were 4 abbreviated versions that were essentially motivated by the unidimensional representativeness of the items and the similarity of the factor loadings. On the other hand, the predominant analysis strategies did not consider the items as categorical variables and therefore used estimators for normally distributed continuous variables [i.e., maximum likelihood (ML)]. This may lead to a degree of non-ignorable underestimation of loadings and interfactor correlations, as is usual with CFA and ML estimators (105, 106), even with robust modifications for ML (107). This potential problem was also noted by Higgins et al. (96). It is apparent that, without adjustments or the use of polychoric correlations, factor loadings and correlations may have non-ignorable biases (106). Finally, total scores were obtained in several studies, even though the multidimensional model was advocated and established, which suggested that the MOS construct is represented with several obtainable scores, but not a single global score. A contrast with the rest of the studies was established with Margolis et al. (79), one of the few studies that, as an argument for their study, explicitly acknowledged the highly inconsistent internal structure of the MOS found in preceding studies. This study represented a methodological advance in the evaluation of the structure of the MOS-SSS because it used a recommended methodology for categorical variables [similar to Higgins et al. (96)] and included the comparison of models, including the bifactor model. Their bifactor model did not converge properly (negative variance), and it was concluded that the MOS-SSS model can be represented by a single dimension with numerous correlated errors. These latter findings on the dimensionality of the MOS-SSS, specifically the probable predominant unidimensionality, require careful examination for proper interpretation of its scores.

Given the background set of MOS-SSS validation studies in different cultural groups, the trends in the reporting of the results, and the results obtained, the present study aligns with what was expressed by Stewart and Napoli-Springer (108) and emphasized by Margolis et al. (79), which is the need to reevaluate a measure when the inconsistency in its dimensionality is a verifiable feature in the preceding literature. This need is critical to ensure the interpretation of the MOS-SSS measure and to define usable observed scores for theory and practice. In this sense, the objective was to obtain evidence of the internal structure of the MOS-SSS, incorporating a sequence of methodological decisions to define the number of dimensions, the internal validity of its items, and the parsimony of its interpretation by means of a proposed abbreviated version. This objective was also accompanied by other analyses that provided the remainder of validity evidence: measurement invariance (not performed in almost all previous studies), comparison of measurement models, and equivalence between versions of the MOS-SSS (full version vs. new abbreviated version).

## 2. Materials and methods

The type of study was non-experimental and cross-sectional, and the participants were chosen using non-probabilistic, convenience-based sampling method.

### 2.1. Participants

A total of 470 patients with chronic diseases participated (women: 297, 63.2%; men: 173, 36.8%) with an average age of 51.51 years (SD = 15.45). The participants were interviewed in a family medicine unit in Mexico City. The inclusion criteria were (a) affiliated with and receiving regular treatment in the family medicine service for the control of chronic diseases (DM, HAS, chronic renal disease, chronic obstructive pulmonary disease, obstructive sleep apnea syndrome, degenerative osteoarthrosis, cerebral vascular disease, rheumatoid arthritis, cancer, hypothyroidism and epilepsy), (b) at least 20 years of age, (c) male or female, and (d) signed an informed consent form. The exclusion criteria were (a) inability to read and write and (b) refusal to participate in the study. The elimination criteria included (a) partial or incomplete responses to the measurement instruments and (b) having been detected as a potential generator of biased responses. In this patient sample, chronic mental health diseases, as well as, some chronic autoimmune diseases such as Multiple Sclerosis (MS), Systemic Lupus Erythematosus (SLE), Myasthenia Gravis, Inflammatory Bowel Disease (IBD), among others, have a low prevalence; patients with these diagnoses are generally seen at a third level of care; similarly, patients with HIV/AIDS are seen at a second level of care. Consequently, care of these patients at the *primary health care* is infrequent. No patients with these diagnoses were found in the family medicine office during the study period, so they were not included. Finally, patients with Cerebrovascular Disease (CVD) and/or Cerebrovascular Accident (CVA) were included in the study, six patients participated (1.3%).

### 2.2. Ethical considerations

This study is part of the research project HIM/2015/017/SSA.1207 “Efectos del entrenamiento en mindfulness sobre el distrés psicológico y la calidad de vida del cuidador familiar,” which was approved by the Research, Ethics, and Biosafety Committees of the Hospital Infantil de México Federico Gómez, Instituto Nacional de Salud, in Mexico City. To conduct this study, we followed the rules and ethical considerations for human research currently applicable in Mexico (109, 110) and those described in Sociedad Mexicana de Psicología American Psychological Association (111). All patients were informed about the objectives and scope of the research and their rights in accordance with the Declaration of Helsinki (112). Patients who agreed to participate in the study signed a letter of informed consent. Participation in this study was voluntary and did not involve payment.

### 2.3. Procedure

Once the research protocol was approved, the battery of measurement instruments was integrated. The patients were identified by the research team in the waiting rooms and in the consultation room of the family medicine unit. Then, the team members asked the patients for their voluntary participation in the study, and they were presented with the informed consent letter, which they signed. Likewise, they were guaranteed their right to withdraw from the study at any time they wished without an impact on or risk to their care in the institution. The participants were informed about the objective of the research, the instruments they would complete and the time they should have available for this activity. At all times, the interviewer verified that there were no unanswered questions to prevent having missing values. At the end of the interview, the patients were verbally thanked and were given the opportunity to express any doubts or concerns about their participation.

### 2.4. Measures

#### 2.4.1. Medical outcomes study-social support survey (MOS-SSS)

This self-report questionnaire consisted of 20 items rated on a five-point Likert-type scale that ranged from 1 “never” to 5 “always”; the first item reported on the size of the social network, and the subsequent 19 items measured four dimensions of functional social support: emotional/informational support (the expression of positive affect and the provision of advice, information, guidance or feedback) (eight items: 3, 4, 8, 9, 13, 16, 17, and 19), instrumental support (the provision of material or behavioral assistance) (four items: 2, 5, 12, and 15), positive social interaction (the availability of other people to do fun things with you) (four items: 7, 11, 14, and 18) and affective support (including expressions of love and affection) [three items: 6, 10, and 20 (69)]. The present study used the Spanish version from Ahumada et al. (76).

#### 2.4.2. Questionnaire of sociodemographic variables for research on family caregivers of children with chronic diseases (Q-SV)

This questionnaire contained 20 items that collect information on sociodemographic, medical, sociocultural and family variables from families of children with chronic diseases. The content of this questionnaire maximized the amount of demographic information, with content relevant to these families (113).

#### 2.4.3. SF-36 scale of health-related quality of life

This is a Likert-type scale (36 items) that evaluated positive and negative states of physical and mental health; item 2 is a transition item that asks about the change in the general state of health with respect to the previous year and was not used for the calculation of any of the 8 dimensions of health status: physical function (ten items), physical role (four items), bodily pain (two items), general health (five items), vitality (four items), social function (two items), emotional role (three items) and mental health (five items). The reported Cronbach’s alpha reliability coefficient was reported to range from 0.56 to 0.84 for the different dimensions (114).

### 2.5. Data analysis

First, data cleaning focused on the detection of excessively inconsistent and consistent responses, i.e., possible response biases. These response patterns were examined by means of the multivariate distance *D*^2^ (115) and the longest sequence of consecutive responses [longstring; Johnson (116)]. For *D*^2^, the cutoff point for detection was *D*^2^ > 36.19 (at *p* = 0.01); for the longstring method, the cutoff point for detection was half the number of items in the total instrument (117, 118), i.e., 19/2 = 9.5 (set to 10). To reduce false negatives, Tukey’s fences were also used, with parameter k set to 1. The database consisted of retaining participants not detected by the two independent methods. Both methods are recommended for the identification of suspected cases of insufficient effort when answering long questionnaires (119). The analysis was performed with the R program *careless* (120).

With the database without the participants showing potentially biased responses, descriptive and association statistics were obtained for items treated as ordinal categorical variables (121), specifically to identify associations with sex (Glass rank biserial correlation coefficient), chronological age (Spearman’s correlation coefficient), marital status (ordinal eta-squared), and education (ordinal eta-squared). These associations may be potential indicators of the differential functioning of items in the compared groups and of the sensitivity of the content for score interpretation purposes (122). The analysis was performed with the R programs *rcompanion* (123) and *MVN* (124).

To test the internal structure of the instrument, we first applied a non-parametric approach, Mokken scaling analysis (MSA) (125), that is a method focused on the psychometric properties of the observed score by analyzing the number of dimensions, the scaling of items and scores, local independence, and the monotonic item-score relationship (125, 126), as these are characteristics that build the monotonic homogeneity model (MHM) (125). MSA does not require the assumptions of parametric analyses [e.g., structural equation modeling or item response theory; Crişan et al. (127)] and is a preliminary procedure for subsequent latent construct analysis (127, 128). Additionally, this method was considered appropriate given the moderate sample size in each randomly drawn subsample and the small number of items in some of the MOS-SSS subscales. Within the MSA, to determine the number of instrument scales, the automated item selection procedure (AISP) (125, 126) was used with the normal search based on the increasing scalability of items grouped by the scalability coefficient H (127). The analysis was performed with the R program *mokken* (129).

To obtain parametric estimates of the internal structure of the MOS and based on the results of the MSA, parallel analysis (PA) (130) was used to identify the number of latent dimensions, and confirmatory factor analysis of structural equation modeling (CFA-SEM) was used to contrast measurement models. Used on categorical variables, such as MOS-SSS items, PA is still an optimal method for estimating the number of latent dimensions (131). PA was used on the interitem polychoric correlations of the simulated data in PA using the psych program (132). The total sample was divided into two halves to assess the replicability of the number of dimensions.

With CFA-SEM, we evaluated (a) the 4-factor multidimensional model of Ahumada et al. (76), which was the source of the MOS-SSS version used in this study, and (b) the unidimensional model, whose result was obtained from the MSA. The weighted least square mean and variance adjusted (WLSMV) estimator was used on the interitem polychoric correlations, given that the items were treated as categorical variables (133). Fitting of these models was performed with approximate fit indices, such as CFI (>0.95), RMSEA (<0.05), and SRMR (<0.05). The 4-factor model was further evaluated on its discriminative validity among its factors with the heterotrait-monotrait ratio (HTMT) criterion (134, 135), a measure sensitive to the degree of statistical differentiation in SEMs (136–138). HTMT compares interitem correlations of different constructs (heteroattribute – heteromethod correlations) with interitem correlations of the same construct (monoattribute – heteromethod correlations). Two cutoff points were chosen, namely, HTMT > 0.90 (139) and HTMT > 0.85 (137, 138, 140), to identify factors with poor discriminative validity and moderate discriminative validity, respectively.

The dimensionality results of the MOS-SSS were assessed for replicability by randomly partitioning the sample into two halves, *n* = 159 and *n* = 158 (this was done on the total clean sample, *n* = 317).

Based on the results of the internal structure, an abbreviated version was created (MOS-S) from the internal strength of the scale, which retained more construct variance (141, 142). Thus, 2 items with the highest factor loadings were chosen in the unidimensional factor solution, but each item also corresponded with each theoretical dimension. For items with equal loadings, the content was considered to maximize content heterogeneity and was chosen for the short version. Equivalence between the short and long versions was assessed by linear correlation (142) but with correction for overlap (143), which is especially used to remove correlated error variance when both versions come from the same administered group (141). Equivalence was further assessed with the classificatory agreement generated by both scores at levels of 3 (tertiles), 4 (quartiles) and 5 groups (quintiles); the coefficient of agreement AC (144) was used.

Measurement invariance of the MOS-S was evaluated with respect to the sex group of the patients. Taking into account the sample size of the study [>300; Chen (145)], the suggested invariance criteria for CFI, SRMR, and RMSEA were <0.010, <0.030, and <0.015, respectively (145). Participant sex was chosen as a possible source of measurement invariance because this phenotypic characteristic is usually included in studies of invariance of psychosocial measures (122).

The reliability of the scores of the final version of the MOS-SSS was estimated with the omega coefficients for categorical variables (146) and with the Molenaar-Sijtsma coefficient (MS-rho) (147), both from SEM and MSA modeling, respectively. The alpha coefficients were also estimated. These estimates were made with the R programs *mokken* (129) and *MBESS* (148).

Finally, as evidence of the relationship between the construct measured by both versions of the MOS and the dimensions of the SF-36, correlation analyses were performed using Pearson’s linear association coefficient. The difference in the correlations obtained between each of the versions of the MOS and the SF-36 was evaluated with Hittner et al.’s (149) z test and the confidence interval for the difference between dependent correlations (150). The procedure was performed with the R program cocor (151).

## 3. Results

### 3.1. Sociodemographic and clinical characteristics of patients with chronic disease

The results indicated that most of participants were female (63.2%) and the average age was 51.51 years (SD = 15.45); the three most prevalent diseases were HAS (63%), DM (56.4%) and musculoskeletal diseases (19.1%); and the total number of diseases experienced by a patient ranged from a minimum of 1 to a maximum of 5 diseases (*M* = 1.63, SE = 0.76). In most cases, high school was the highest level of education attained by the participants (26%). A high percentage of the patients were married (55.5%), and the majority reported a monthly income of between $2,700 and $6,799 (53.2%). This information can be seen in Table 2.

**TABLE 2:** Sociodemographic and clinical variables characteristics of patients with CDs (*n* = 470).

	*N*	%	*M*	SD
**Sex**				
Female	297	63.2		
Male	173	36.8		
**Age**				
20–29	47	10		
30–39	71	15.1		
40–49	100	21.3		
50–59	88	18.7		
60–69	108	23		
70–79	45	9.6		
80–89	10	2.1		
90–99	1	0.2		
Total	–	–	51.51	15.45
**Marital status**				
Married	261	55.5		
Single	67	14.3		
Widowed	38	8.1		
Divorced	22	4.7		
Free-union	82	17.4		
**Instruction**				
Primary incomplete	35	7.4		
Primary complete	83	17.7		
Secondary incomplete	15	3.2		
Secondary complete	122	26		
High school incomplete	6	1.3		
High school complete	86	18.3		
Technical	49	10.4		
Bachelor	52	11.1		
Graduate	1	0.2		
No studies	21	4.5		
**Monthly income (Mexican currency)**				
0–2,699	56	11.9		
2,700–6,799	250	53.2		
6,800–11,599	120	25.5		
11,600–34,999	44	9.4		
**Disease**				
HAS	296	63		
DM	265	56.4		
CKD	14	3		
COPD	22	4.7		
OSA	10	2.1		
AMI	14	3		
CVD	6	1.3		
Osteomuscular diseases	90	19.1		
RA	5	1.1		
Cancer	19	4		
Hypothyroidism	20	4.3		
Epilepsy	6	1.3		

CDs, chronic diseases; HAS, systemic arterial hypertension; DM, diabetes mellitus; CKD, chronic kidney disease; COPD, chronic obstructive pulmonary disease; OSA, obstructive sleep apnea syndrome; AMI, acute myocardial infarction; CVD, cerebral vascular disease; RA, rheumatoid arthritis.

### 3.2. Possible response biases

Seventy-nine cases were detected with the *D*^2^ method, with an inconsistent response pattern in the set of 19 items (16.8%); with the longstring method, consecutive identical responses had a median of 5 consecutive identical responses (*M* = 7.6, Q1 = 4, Q3 = 10) and a range between 2 and 19 identical responses. Applying Tukey’s fences criterion (longstring > 16), 74 (15.7%) cases were detected between 17 and 19 consecutive identical responses. The linear correlation between the two estimators was high (*r* = −0.58, *p* < 0.01, 95% CI = −0.64, −0.52; for the classification of detected cases: Cramer *V* = 1.0, 95% CI = 0.98, 1.00, χ^2^ = 7,520.0, *p* < 0.01, gl = 4,672). Because of this high divergence, no subjects were detected with both methods; the detected cases were excluded (79 and 74, 32.5%), and the sample for analysis consisted of 317 participants (78.9%).

### 3.3. Descriptive, normality and association statistics for the MOS items

The mean responses on the items tended to be similar since all were >3.0 (Table 3). The highest mean response (4.00) was only 0.29 times higher than the lowest mean response (3.1). The above pattern of similarity was also observed in the dispersion of the items estimated by the standard deviation of each item, where the maximum (1.49) and minimum (1.24) mean dispersion was only 0.20 times. Regarding the distribution of the items in the range of response options, skewness and kurtosis were in the same direction (i.e., negative), suggesting a highly similar distributional behavior. In the same line of results, univariate normality did not hold for all items.

**TABLE 3:** Descriptive and association statistics for MOS-SSS/MOS-S items (*n* = 317).

	Descriptive					Association			
	*M*	SD	Sk	*K*	AD	Sex	Age	Marital	Instruct.
MOS3	3.61	1.29	−0.52	−0.88	15.28	0.06	−0.02	−0.00	−0.01
MOS4	3.48	1.32	−0.40	−1.00	13.12	0.12	−0.01	−0.00	−0.00
MOS8	3.57	1.34	−0.62	−0.83	16.33	0.03	0.06	−0.00	0.00
MOS9	3.62	1.34	−0.58	−0.95	17.50	0.13	0.00	−0.00	0.00
MOS13	3.41	1.42	−0.39	−1.20	15.09	0.10	−0.03	−0.00	0.00
MOS16	3.33	1.43	−0.23	−1.33	14.67	0.06	−0.00	0.00	−0.00
MOS17	3.34	1.41	−0.25	−1.29	14.03	0.11	−0.03	−0.00	0.00
MOS19	3.56	1.32	−0.46	−0.98	14.74	0.07	0.01	−0.00	−0.01
MOS2	3.10	1.49	−0.15	−1.42	14.37	0.08	0.04	−0.00	−0.01
MOS5	3.48	1.48	−0.46	−1.24	18.93	0.07	0.07	−0.00	−0.00
MOS12	3.58	1.46	−0.54	−1.12	2.85	−0.01	0.03	0.00	−0.01
MOS15	3.47	1.40	−0.44	−1.14	15.76	0.02	0.02	0.00	−0.01
MOS7	3.88	1.24	−0.87	−0.32	21.70	0.07	0.00	0.00	−0.00
MOS11	3.57	1.38	−0.46	−1.11	17.49	0.06	0.02	−0.00	−0.00
MOS14	3.53	1.32	−0.35	−1.20	15.99	0.08	0.03	−0.00	0.00
MOS18	3.60	1.27	−0.45	−0.97	14.90	0.04	−0.08	−0.00	−0.01
MOS6	4.00	1.25	−1.09	0.01	28.71	0.10	0.04	−0.00	−0.00
MOS10	3.77	1.42	−0.75	−0.84	25.94	0.15	0.01	−0.00	0.00
MOS20	3.81	1.32	−0.71	−0.79	23.26	0.10	0.01	−0.00	−0.00

Sk, skew coefficient; K, kurtosis coefficient; AD, Anderson–Darling normality test; Marital, marital status; Instruc., instruction level.

### 3.4. Associations between the MOS items and demographic variables

Regarding the association of the MOS items with demographic variables (Table 3), the association with sex (min = −0.01, max = 0.15, Md = 0.07), chronological age (min = −0.08, max = 0.07, Md = 0.01), marital status (min = 0.00, max = 0.00, Md = 0.00), and education (min = −0.01, max = 0.00, Md = 0.00) were all maintained around zero, and there was an absence of statistical significance.

### 3.5. Evidence of the internal structure of the MOS social support survey

#### 3.5.1. Non-parametric modeling (Mokken scaling analysis)

Table 4 shows the results of the MSA modeling. The AISP algorithm for Mokken scale selection yielded a likely different MOS structure. At the 0.30, 0.40, and 0.50 levels of the scaling coefficient H, the number of scale differences remained constant, where a single dimension was the apparent best definition of the internal structure of the MOS. This result was replicated in the two samples randomly drawn from the total sample, indicating that the unidimensionality of the MOS was replicable.

**TABLE 4:** MSA: Number of dimensions (AISP) and monotonic homogeneity model (MHM).

	Total sample (*n* = 317)					Random sample 1 (*n* = 159)					Random sample 2 (*n* = 158)				
	AISP			MHM		AISP			MHM		AISP			MHM	
	0.3	0.4	0.5	H	Crit	0.3	0.4	0.5	H	Crit	0.3	0.4	0.5	H	Crit
MOS3	1	1	1	0.72	0	1	1	1	0.68	0	1	1	1	0.75	0
MOS4	1	1	1	0.69	0	1	1	1	0.67	0	1	1	1	0.70	0
MOS8	1	1	1	0.76	0	1	1	1	0.73	0	1	1	1	0.79	0
MOS9	1	1	1	0.77	0	1	1	1	0.74	0	1	1	1	0.80	0
MOS13	1	1	1	0.74	0	1	1	1	0.72	0	1	1	1	0.77	0
MOS16	1	1	1	0.78	0	1	1	1	0.73	0	1	1	1	0.82	0
MOS17	1	1	1	0.76	0	1	1	1	0.71	0	1	1	1	0.80	0
MOS19	1	1	1	0.73	0	1	1	1	0.68	0	1	1	1	0.79	0
MOS2	1	1	0	0.46	38	1	0	0	0.39	0	1	1	1	0.52	9
MOS5	1	1	1	0.66	0	1	1	1	0.64	0	1	1	1	0.68	0
MOS12	1	1	1	0.69	0	1	1	1	0.66	0	1	1	1	0.71	0
MOS15	1	1	1	0.70	0	1	1	1	0.66	0	1	1	1	0.73	0
MOS7	1	1	1	0.75	0	1	1	1	0.76	0	1	1	1	0.75	0
MOS11	1	1	1	0.75	0	1	1	1	0.72	0	1	1	1	0.79	0
MOS14	1	1	1	0.75	0	1	1	1	0.73	0	1	1	1	0.77	0
MOS18	1	1	1	0.70	0	1	1	1	0.66	0	1	1	1	0.75	0
MOS6	1	1	1	0.76	0	1	1	1	0.77	0	1	1	1	0.76	0
MOS10	1	1	1	0.76	0	1	1	1	0.75	0	1	1	1	0.78	0
MOS20	1	1	1	0.62	0	1	1	1	0.68	0	1	1	1	0.64	0

MSA, Mokken scaling analysis; AISP, automated item selection procedure; MHM, monotonic homogeneity model.

#### 3.5.2. Parametric modeling (–CFA-SEM)

Given the results of the non-parametric modeling, where the apparent unidimensionality can be accepted, the dimensionality was again examined using linear parametric modeling. Table 5 shows the results of the PA on the total sample and on the two randomly drawn samples. The calculated eigenvalues clearly differentiated between a model possibly represented by a single factor (eigenvalues > 11.00) compared to the dimensionality of two or more factors (eigenvalues < 1.00). The corresponding graphs in each analysis also show the representativeness of a single dominant factor and its replicability.

**TABLE 5:** Parallel analysis (number of factors).

Total sample (*n* = 317)	*N* factors	Eigenvalues	
		Real	Simulated
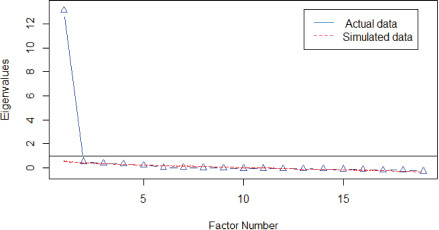
	1	13.12	0.58
	2	0.52	0.39
	3	0.35	0.33
	4	0.31	0.27
	5	0.19	0.22
**Random sample 1 (*n* = 159)**	***N* factors**	**Real**	**Simulated**
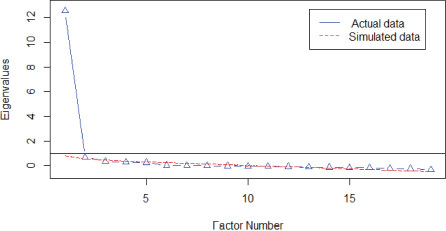
	1	12.54	0.81
	2	0.68	0.54
	3	0.34	0.46
	4	0.27	0.38
	5	0.24	0.30
**Random sample 2 (*n* = 158)**	***N* factors**	**Real**	**Simulated**
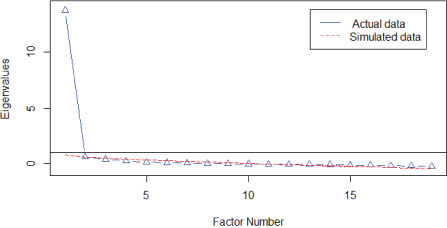
	1	13.70	0.77
	2	0.59	0.56
	3	0.36	0.47
	4	0.23	0.38
	5	0.11	0.30

The final decision regarding dimensionality was evaluated in the total sample, with the comparative fit of two models, one representing the 4-factor multidimensional model [from Ahumada et al. (76)] and the unidimensional model (suggested in the previous sections of the present study). The fit of the 4-factor multidimensional model (MOS-4F) revealed WLSMV-χ^2^ = 332.745 (df = 146), CFI = 0.999, RMSEA = 0.064 (90% CI = 0.055, 0.073), and SRMR = 0.039. The unidimensional model (MOS-1F) also showed an acceptable fit with WLSMV-χ^2^ = 509.44 (df = 171), CFI = 0.998, RMSEA = 0.086 (90% CI = 0.078, 0.095), and SRMR = 0.048. The factor loadings obtained in both models were high (>0.60); although the RMSEA indicated that the degree of misfit was lower in the multidimensional model compared to the unidimensional one (RMSEA_*MOSS–*4*F*_ < RMSEA_*MOSS–*1*F*_), it was observed that there was no substantial difference in the fit indices between the two models.

A comparative inspection in detail of the obtained parameters (i.e., factor loadings and interfactor correlations; Table 6) revealed that the size of the factor loadings was highly similar (*r* = 96, *p* < 0.01; congruence coefficient = 0.99). Additionally, the correlations between factors ranged between 0.99 and0.86, a size range that can be considered high (137). Assessment of the discriminative validity between factors (under the heading “Correlations/HTMT” in Table 6) yielded HTMT indices that essentially bordered on or exceeded both criteria for poor discrimination (HTMT ≥ 0.94). Given the results of both MSA and CFA-SEM analyses, the unidimensional model appears to represent social support well, without loss of internal validity in the present sample.

**TABLE 6:** CFA-SEM of the MOS-SSS and MOS-S (*n* = 317).

	MOS–SSS				MOS-1F	MOS-S		
						CFA	MSA	
	EMI	TAN	PSI	AFF			H	Crit
MOS3	0.86				0.85	–	–	–
MOS4	0.84				0.83	–	–	–
MOS8	0.93				0.92	–	–	–
MOS9	0.94				0.93	0.83	0.80	0
MOS13	0.92				0.92	–	–	–
MOS16	0.95				0.94	0.92	0.81	0
MOS17	0.94				0.93	–	–	–
MOS19	0.91				0.90	–	–	–
MOS2		0.60			0.55	–	–	–
MOS5		0.87			0.79	–	–	–
MOS12		0.92			0.85	0.88	0.74	0
MOS15		0.92			0.85	0.87	0.75	0
MOS7			0.91		0.91	–	–	–
MOS11			0.92		0.92	0.93	0.81	0
MOS14			0.91		0.91	0.90	0.79	0
MOS18			0.86		0.86	–	–	–
MOS6				0.94	0.92	0.89	0.78	0
MOS10				0.95	0.92	0.94	0.81	0
MOS20				0.78	0.76	–	–	–
**Correlations/HTMT**								
	**EMI**	**TAN**	**PSI**	**AFF**				
EMI	1	0.87	0.98	0.91				
TAN	0.88	1	0.86	0.84				
PSI	0.99	0.88	1	0.97				
AFF	0.94	0.86	0.99	1				

MOS-SSS, 19 items MOS-SSS; EMI, emotional/informational support; TAN, tangible support; AFF, affective support; PSI positive social interaction; HTMT, heterotrait-monotrait correlation. MOS-1F, unidimensional model; MOS-S, final model for MOS-S (8 items); CFA, CFA-SEM; MSA, Mokken scaling analysis; H, *scalability coefficients*; Crit weighted criterion for the monotonic homogeneity model.

#### 3.5.3. Short version (MOS-S)

The items with the highest factor loadings in their previous content dimensions were as follows (Table 6): 9, 16, 17, 12, 15, 7, 11, 14, 6 and 10. Based on the content analysis, the content of item 17 can be subsumed in item 9, where sharing and expressing concerns can be oriented to several purposes, among them, problem solving. Item 14 seemed more directly linked to the content of the rest of its theoretical dimension because of the reference to the condition of health or illness. The final short version consisted of eight items: 9, 16, 12, 15, 7, 11, 6 and 10. Table 6, under the heading “short version,” shows the recalculated parameters for the items of this abbreviated version, with CFA-SEM and MSA. Strong factor loadings are observed (>0.81) and are similar to their corresponding factor loadings in the long version (congruency coefficient = 0.99).

#### 3.5.4. Measurement invariance

The configurational, metric and scalar invariances were satisfactory (Table 7). Additionally, the differences between these models indicated that the invariance in the psychometric parameters of the MOS-S was maintained up to the invariance in the residuals. Based on these results, the parameters obtained in the total sample are equally representative for both sex groups of patients.

**TABLE 7:** MOS-S measurement invariance (group = sex).

	Configurational	Metric	Intercepts	Residuals
**Fit measures**				
WLSMV-x2 (df)	83.83** (40)	106.07** (63)	118.68** (70)	118.68** (78)
CFI	0.999	0.999	0.999	0.999
SRMR	0.038	0.039	0.039	0.039
**Differences**				
Δ_*CFI*_	–	0.001	0.00	
Δ_*SRMR*_	–	0.000	0.00	0.00

MOS-S, short version of MOS-SSS. **p < 0.01.

### 3.6. Reliability

The reliability of the score from the new abbreviated version of the MOS was α = 0.95 (95% CI = 0.94, 0.96) and ω = 0.97 (95% CI = 0.97, 0.99); based on the MSA framework, the reliability was rho-MS = 0.96. The standard error of measurement (using SD in the total sample = 9.75, and the alpha coefficient) corresponding to this score was 2.18. The reliability of the long version of the MOS, with a single score, was α = 0.99 (95% CI = 0.99, 1.00) and ω = 0.99 (95% CI = 0.99, 1.00). The difference between the internal consistency of the shortened version and the unidimensional long version can be considered trivial.

### 3.7. Equivalence between versions (MOS-SSS and MOS-S)

The linear association between the scores of both unidimensional short and long versions was *r* = 0.98 (*t* = 95.3, df = 315, *p* < 0.01); with correction for overlapping, the correlation was 0.95. The degree of agreement (Gwet’s AC1 coefficient) between the classification of scores into tertiles, quartiles, and quintiles produced by both scores (short and long-unidimensional version) was, respectively, AC1 = 0.90 (*p* < 0.01; 95% CI = 0.86, 0.94), AC1 = 0.86 (*p* < 0.01; 95% CI = 0.82, 0.90), and AC1 = 0.80 (*p* < 0.01; 95% CI = 0.75, 0.85).

### 3.8. Association with other variables

The linear association of both versions of the MOS (19-item and 8-item versions) is shown in Table 8. Except for physical role, the rest of the correlations were statistically non-significant and practically zero. Statistical comparison between the two versions of the MOS indicated an absence of substantial differences, and differences that were rather trivial in size. Although a statistically significant difference was found (in social function), the size of this difference can be considered trivial (see the range of the difference in these correlations, Δ_*r*_).

**TABLE 8:** Association with other variables: comparison MOS-SSS vs. MOS-S.

	MOS-SSS (19 items)	MOS–S	*Z*_*HMS*_	95% CI Δ_*r*_
**SF-36**				
Physical functioning	0.154	0.150	0.38	−0.01, 0.02
Role limitations due to physical health	0.19*	0.19*	−0.35	−0.02, 0.01
Pain	−0.15	−0.15	0.78	−0.01, 0.02
General health	0.01	0.02	0.96	−0.01, 0.03
Energy/fatigue	−0.03	−0.03	−0.11	−0.02, 0.01
Social functioning	−0.03	−0.06	2.86*	0.00, 0.04
Role limitations due to emotional problems	0.11	0.16	−0.44	−0.02, 0.01
Emotional well-being	−0.00	0.00	−0.46	−0.02, 01

*Z*_*HMS*_: Hittner et al.’s (149) *z*-test. Δ_*r*_: 95% confidence interval for difference. *p < 0.006 (nominal alpha with Bonferroni’s correction: 0.05/8 scores = 0.006).

## 4. Discussion

The objectives of this research were to obtain evidence of the validity of the MOS scale with respect to its factorial structure, its internal consistency reliability, and its relationship with other variables. The complementary objectives were to describe the distribution of its scores. This study was implemented in patients with chronic disease at the primary health care, where the measurement of social support is relevant for knowing the resources that can impact the patient’s quality of life.

According to the results, the unidimensional model adequately represents the construct of social support measured by the MOS. The validity of the items with respect to their latent constructs was not affected by the shift from multidimensional modeling to the unidimensional model. One implication of this is that social support represented by a single score does not alter the significance of the items in defining an overall construct. However, another implication is that the items do not represent content that previously appeared to be differentiated, i.e., the items do not represent specific dimensions such as the four dimensions obtained in Ahumada et al. (76). This unidimensional representation of the construct measured by the MOS leads to rethinking the theoretical definition of social support coming from the MOS framework, as well as testing a definition for the interpretation of the total score of the instrument. This definition is more parsimonious since it is focused on a general domain and not divided into separate dimensions.

The results are not congruent with the conclusions of the Hispanic studies (see Table 1), including those reported by Sherbourne and Stewart (69), because these studies reported the apparent multidimensionality of the MOS. As described in the Introduction, this discrepancy is fueled by the methodological characteristics of these studies that influenced decision-making about internal structure, as well as by the incomplete reporting of their factorial results. Specifically, few of these studies reported interfactor correlations [e.g., (78, 83)], and when reported, the size of the interfactor correlations showed a range between 0.59 and 0.75 (78) or 0.97 and 0.99 (83). These magnitudes are clearly high or very high and show that the discriminative validity of the MOS scales is not defensible and that a unidimensional factorial solution may be the best representation of the construct. This problem in discriminative validity was also reported in the MOS creation study, in which the relationship between emotional and informational support was 0.99 (69), and the unhypothesized item-scale correlations studied were approximately 0.50. On the other hand, in other studies, the degree of discriminative validity could be assessed because the analytic strategy forced us to estimate the interfactor correlation (varimax rotation), or it was not reported. Along with this type of orthogonal rotation, in which the factors are assumed to be completely independent, the analysis of principal components and the number of dimensions using the Kaiser criterion (eigenvalue > 1) were also frequent. This package of methodological choices is known as the little jiffy (152).

Another reason for divergence was that several studies reported 2 and 3 factors (77, 86, 88, 90, 98). However, these studies did not report interfactor correlations and/or did not compare measurement models (e.g., unidimensional or bifactor models), and it is difficult to be sure whether a single dimension competed with the multidimensionality found in these studies. However, regarding the study by Margolis et al. (79), our study found partial convergence, given that they concluded unidimensionality but with the addition of correlated errors and high factor loadings on the global factor. In the present study, the high psychometric similarity of the 19 items was considered a strong justification to produce a shortened version and to avoid the occurrence of correlated errors and maximize the parsimonious measurement of the MOS.

The present study also made progress in generating an abbreviated measure, given that a) factor loadings were highly similar in the unidimensional solution, and therefore the construct validity of the items did not differentiate between items that may have been more valid than others; b) an abbreviated measure is parsimonious to interpret, and c) this may be an important opportunity for choosing between screening measures or lengthy community surveys. This result adds to the existing abbreviated versions and may provide an equivalent measure of social support as these measures, given that the items are psychometrically similar with respect to their overall construct, social support. However, a comparative evaluation of these short versions with respect to subject classification and association with external variables is needed. Because previous brief versions were generated from models with different numbers of factors and an emphasis on tangible support [e.g., (73, 91)] or different samples of participants [e.g., mothers of children in clinical treatment; Gjesfjeld et al. (85)], the version obtained here may be more appropriate for the study sample. Given the strength of the validity of the items in their single dimension, it is likely that this version is generalizable to other groups of participants, but this assertion is conditional on future studies.

In the analysis of the equivalence between the unidimensional score with the 19 items and the abbreviated version, the high linear correlation between the two versions of the instrument indicates that the scaling of people based on the scores would be practically equal and that both scores can be used equivalently to differentiate the magnitude of perceived social support. When people are classified ordinally into groups of 3, 4 or 5 clusters, the agreement was also somewhat high, although it was higher in the tercile classification (i.e., low, medium, high), which suggested that the classification will be more equivalent between both MOS-SSS and MOS-S scores with fewer clusters. In summary, the analysis of the equivalence of the two versions of the MOS for differentiating subjects using direct scores or rankings (i.e., based on tertiles, quartiles, or quintiles) is highly similar. This high similarity is associated with the high coefficient of consistency obtained with both scores because it indicated that the error variance is very small, and the variability around the direct score will not produce severe changes in the description of the person assessed.

This level of reliability may indicate that the MOS score is useful in clinical practice, where individual decisions require highly accurate measures, i.e., with as little error variability as possible. Given that there appears to be no substantial loss of accuracy, according to the results of the equivalence between scores and internal consistency, the use of the abbreviated version is recommended for screening and clinical assessment purposes; specifically, for individual descriptions related to the diagnosis and psychosocial variables derived from social support, for individual reports on the patient’s social support status, and for making individual decisions on personalized interventions. Another implication of the obtained reliability results is that there was a strong replicability of the scores in a hypothetical situation where the MOS-S measurement is applied repeatedly. This indicates that the degree of error is small and advisable for clinical purposes because a reliability coefficient > 0.90 implies little probability of measurement error when applied for decision making on individual examinees. This is especially useful in individual interventions.

The association of the MOS with the SF-36 yielded low linear dependence, indicating divergence between the constructs assessed by these measures but also the possible specificity of these scores in this participant sample. In this sense, the physical role score was comparatively more strongly associated with the MOS, and it is very consistent with this research, given the basic characteristics of the sample. The study sample comprised patients with chronic diseases, and given the specific condition and severity of the disease, these patients will require support for roles that require moderate or intense physical exertion. In this sense, the new version has potential usefulness in the context of the importance of measuring social support for patients, since it has been shown that social support is an important determinant of physical and mental health because it moderates the effects of stress, improves the well-being of people, and has effects that extend to their family, social and work environment (16, 26–30, 38).

Among the limitations of this study, we can identify the use of non-probabilistic sampling so that population representativeness is not guaranteed. A second limitation is the cross-sectional design, which does not allow us to estimate the temporal reliability or to test the temporal stability of the factor model. A possible limitation is that participants with valid responses (i.e., false positives) may have been included in the removed group because of possible response bias. As a balance to this problem, we used two accepted methods (116–118) that detected two distinct patterns usually associated with possible response insufficiency/bias: extreme consistency (longstring) and inconsistency (outliers). A qualitative examination of this selection, and a sensitivity analysis, can verify whether the detection was correct and its impact large. But surely, some detection is preferable to none. Finally, the relationship with convergent measures of social support was not included, so this source of validity should be included in future studies. As a final note, replication of this work in future studies will allow more precise conclusions to be drawn regarding the factor structure of the MOS scale in patients with chronic disease at the primary health care. In addition, it will be possible to establish the relationship between social support and the degree of severity of chronic diseases and to carry out predictive studies between social support and the severity of chronic diseases in patients being attended in primary health care.

## 5. Conclusion

Due to the multiple clinical implications of social support in patients with chronic disease, the high global and national prevalence of these diseases, most of which are treated at the primary health care, and the instability of the internal structure of the MOS-SSS, the validity of this scale in patients with chronic disease was studied. Based on the results obtained in this study, a unidimensional representation of all MOS items was obtained. Since the items were psychometrically similar, a new 8-item, unidimensional, highly reliable, abbreviated version with invariant structure in the sex group of the patients was developed. This version showed adequate psychometric properties in patients with chronic disease at the primary health care.

## Data availability statement

The original contributions presented in this study are included in this article/supplementary material, further inquiries can be directed to the corresponding author.

## Ethics statement

This study involving human participants was reviewed and approved by the Research, Ethics, and Biosafety Committees of the Hospital Infantil de México Federico Gómez, Instituto Nacional de Salud, in Mexico City. Patients who agreed to participate in the study signed a letter of informed consent.

## Author contributions

MÁNB and JGE: conceptualization. CM-S, MTD-G, MA-R, and FT-T: methodology. MA-R, OAFL, and MÁNB: validation. CM-S and JMR: formal analysis. MÁNB, CM-S, FT-T, and MA-R: investigation. LR-R and FT-T: resources. MTD-G, JMR, and GH-S: data curation. JHRC, FT-T, and OIGP: writing—original draft preparation. FT-T, MD-G, CM-S, GH-S, MA-R, and JHRC: writing—review and editing. CIA-G and LR-R: visualization. AL-L, CIA-G, and JM-R: supervision. JGE and FT-T: project administration and funding acquisition. All authors have read and agreed to the published version of the manuscript.
